# Maternal mortality and morbidity burden in the Eastern Mediterranean Region: findings from the Global Burden of Disease 2015 study

**DOI:** 10.1007/s00038-017-1004-3

**Published:** 2017-08-03

**Authors:** GBD 2015 Eastern Mediterranean Region Maternal Mortality Collaborators, Ibrahim Khalil, Michael Collison, Charbel El Bcheraoui, Raghid Charara, Maziar Moradi-Lakeh, Ashkan Afshin, Adrienne Chew, Farah Daoud, Kristopher J. Krohn, Danny Colombara, Rebecca Ehrenkranz, Michael Kutz, Haidong Wang, Amanuel Alemu Abajobir, Foad Abd-Allah, Haftom Niguse Abraha, Laith J. Abu-Raddad, Aliasghar Ahmad Kiadaliri, Alireza Ahmadi, Kedir Yimam Ahmed, Muktar Beshir Ahmed, Faris Hasan Al Lami, Khurshid Alam, Deena Alasfoor, Reza Alizadeh-Navaei, Juma M. Alkaabi, Fatma Al-Maskari, Rajaa Al-Raddadi, Khalid A. Altirkawi, Nahla Anber, Hossein Ansari, Hamid Asayesh, Rana Jawad Asghar, Tesfay Mehari Atey, Tadesse Awoke Ayele, Till Bärnighausen, Umar Bacha, Aleksandra Barac, Suzanne L. Barker-Collo, Bernhard T. Baune, Shahrzad Bazargan-Hejazi, Neeraj Bedi, Isabela M. Bensenor, Adugnaw Berhane, Addisu Shunu Beyene, Zulfiqar A. Bhutta, Dube Jara Boneya, Rohan Borschmann, Nicholas J. K. Breitborde, Zahid A. Butt, Ferrán Catalá-López, Liliana G. Ciobanu, Hadi Danawi, Amare Deribew, Samath D. Dharmaratne, Kerrie E. Doyle, Aman Yesuf Endries, Emerito Jose Aquino Faraon, André Faro, Maryam S. Farvid, Wubalem Fekadu, Seyed-Mohammad Fereshtehnejad, Florian Fischer, Tsegaye Tewelde Gebrehiwot, Ababi Zergaw Giref, Melkamu Dedefo Gishu, Alessandra Carvalho Goulart, Tesfa Dejenie Habtewold, Randah Ribhi Hamadeh, Mitiku Teshome Hambisa, Samer Hamidi, Josep Maria Haro, Mohammad Sadegh Hassanvand, Nobuyuki Horita, Mohamed Hsairi, Hsiang Huang, Abdullatif Husseini, Mihajlo B. Jakovljevic, Spencer Lewis James, Jost B. Jonas, Amir Kasaeian, Yousef Saleh Khader, Ejaz Ahmad Khan, Abdullah Tawfih Abdullah Khoja, Ardeshir Khosravi, Jagdish Khubchandani, Daniel Kim, Yun Jin Kim, Yoshihiro Kokubo, Ai Koyanagi, Barthelemy Kuate Defo, Heidi J. Larson, Asma Abdul Latif, Paul H. Lee, Cheru Tesema Leshargie, Ricky Leung, Loon-Tzian Lo, Raimundas Lunevicius, Hassan Magdy Abd El Razek, Mohammed Magdy Abd El Razek, Reza Majdzadeh, Azeem Majeed, Reza Malekzadeh, Jose Martinez-Raga, Habibolah Masoudi Farid, Mohsen Mazidi, John J. McGrath, Ziad A. Memish, Walter Mendoza, Melkamu Merid Mengesha, Mubarek Abera Mengistie, George A. Mensah, Haftay Berhane Mezgebe, Ted R. Miller, Philip B. Mitchell, Alireza Mohammadi, Shafiu Mohammed, Carla Makhlouf Obermeyer, Felix Akpojene Ogbo, Elizabeth Palomares Castillo, Christina Papachristou, Scott B. Patten, George C. Patton, Pereira M. David, Aslam Pervaiz, Michael Robert Phillips, Farshad Pourmalek, Mostafa Qorbani, Amir Radfar, Anwar Rafay, Vafa Rahimi-Movaghar, Rajesh Kumar Rai, David Laith Rawaf, Salman Rawaf, Amany H. Refaat, Satar Rezaei, Mohammad Sadegh Rezai, Gholamreza Roshandel, Mahdi Safdarian, Mahdi Safiabadi, Saeid Safiri, Rajeh Sagar, Mohammad Ali Sahraian, Payman Salamati, Abdallah M. Samy, Benn Sartorius, Mete I. Saylan, Soraya Seedat, Sadaf G. Sepanlou, Masood Ali Shaikh, Morteza Shamsizadeh, Diego Augusto Santos Silva, Jasvinder A. Singh, Badr H. A. Sobaih, Chandrashekhar T. Sreeramareddy, Dan J. Stein, Rizwan Suliankatchi Abdulkader, Bryan L. Sykes, Rafael Tabarés-Seisdedos, Karen M. Tabb, Arash Tehrani-Banihashemi, Mohamad-Hani Temsah, Abdullah Sulieman Terkawi, Roman Topor-Madry, Kingsley Nnanna Ukwaja, Olalekan A. Uthman, Stein Emil Vollset, Tolassa Wakayo, Yuan-Pang Wang, Andrea Werdecker, Ronny Westerman, Abdulhalik Workicho, Mohsen Yaghoubi, Hassen Hamid Yimam, Naohiro Yonemoto, Mustafa Z. Younis, Chuanhua Yu, Maysaa El Sayed Zaki, Aisha O. Jumaan, Theo Vos, Simon I. Hay, Mohsen Naghavi, Nicholas J. Kassebaum, Christopher J. L. Murray, Ali H. Mokdad

**Affiliations:** Institute for Health Metrics and Evaluation, University of Washington, Seattle, WA USA

**Keywords:** Maternal mortality, Maternal health, Eastern Mediterranean Region, Burden of disease

## Abstract

**Objectives:**

Assessing the burden of maternal mortality is important for tracking progress and identifying public health gaps. This paper provides an overview of the burden of maternal mortality in the Eastern Mediterranean Region (EMR) by underlying cause and age from 1990 to 2015.

**Methods:**

We used the results of the Global Burden of Disease 2015 study to explore maternal mortality in the EMR countries.

**Results:**

The maternal mortality ratio in the EMR decreased 16.3% from 283 (241–328) maternal deaths per 100,000 live births in 1990 to 237 (188–293) in 2015. Maternal mortality ratio was strongly correlated with socio-demographic status, where the lowest-income countries contributed the most to the burden of maternal mortality in the region.

**Conclusion:**

Progress in reducing maternal mortality in the EMR has accelerated in the past 15 years, but the burden remains high. Coordinated and rigorous efforts are needed to make sure that adequate and timely services and interventions are available for women at each stage of reproductive life.

**Electronic supplementary material:**

The online version of this article (doi:10.1007/s00038-017-1004-3) contains supplementary material, which is available to authorized users.

## Introduction

Maternal mortality ratio (MMR), which measures deaths per 100,000 live births, is a standard measure for global, regional, and national comparison (Abouzahir and Wardlaw 2001). It is also one of the main criteria of health outcomes and an indicator of the socioeconomic development level of countries that is recognized worldwide (Liang et al. 2010). Country estimates of maternal mortality over time are crucial to inform the planning of maternal, sexual, and reproductive health programs and to guide advocacy efforts and research at the national level. These estimates are also needed at the international level to inform decision-making concerning resource allocation by development partners and donors (WHO et al. 2007).

In 2015, 275,288 women are estimated to have died as a result of pregnancy or childbirth globally (Kassebaum et al. 2016b). Maternal death is defined by ICD-10 as the death of a woman while pregnant or within 42 days and up to 1 year (late maternal death); of termination of pregnancy, irrespective of the duration and site of the pregnancy, from any cause related to or aggravated by the pregnancy or its management, but not from accidental or incidental causes (World Health Organization 1992).

The primary causes of maternal deaths are hemorrhage, hypertension, infections, pre-existing medical conditions, and lack of literacy, family planning (unmet need and birth spacing), and access to pregnancy termination when needed. Identifying and improving the main factors related to maternal mortality depend on a correct definition of the required priorities for appropriate prevention, diagnosis, and treatment (Ribeiro et al. 2008).

Poverty, illiteracy, malnutrition, and the low social status of women are usually among the major underlying causes of maternal mortality. Analyzing the economic, social, and health system factors affecting maternal mortality can provide some credible information and evidence for public health interventions to improve maternal health. A few general studies on nonclinical determinants of maternal mortality have analyzed different factors including access to safe drinking water, access to food, fertility rate, education level, life expectancy at birth, access to health services, delivery done by skilled birth attendants, out-of-pocket payment, GDP per capita, health expenditures, and government corruption (Hertz et al. 1994; Midhet et al. 1998; Buor and Bream 2004; Alvarez et al. 2009; Muldoon et al. 2011).

From 1990 to 2015, the global MMR decreased 30.6% from 280.4 (95% uncertainty interval (UI): 262.6–299.0)  to 194.7 (172.6–233.2) (Institute for Health Metrics and Evaluation 2017). This decrease was driven in part by the recognition of maternal health as one of the priorities in the UN Millennium Development Goals (MDGs), which provided a strong incentive to reduce MMR by country (Ronsmans and Graham 2006). New goals outlined in the sustainable development goals (SDGs) for 2015 to 2030 build on the momentum of the MDGs and seek to bring the global MMR below 70 deaths per 100,000 live births (United Nations 2015). The greatest challenge in meeting this goal lies with countries in the bottom two quintiles of wealth, where improvement is most needed and has historically occurred slowly if at all (Abouzahir and Wardlaw 2001).

The variation in MMR between countries by income group is drastic, where the burden of maternal mortality falls almost exclusively upon developing countries (Ronsmans and Graham 2006). Globally, the average MMR for countries in the top quintile of wealth for 2015 was 14.5 (13.7–15.4) compared to 440.2 (357.4–543.5) in the bottom quintile (Institute for Health Metrics and Evaluation 2017). Despite this variation, inequalities in maternal mortality exist between and within countries, where mortality in all locations is exacerbated by a lack of access to quality obstetric care, whether because of the remoteness of certain regions or the inability of health facilities to provide proper care (Peterson et al. 2012).

The Eastern Mediterranean Region (EMR) is a diverse region consisting of 22 countries, including high-income countries, where socioeconomic development has progressed considerably over the last decades; middle-income countries that have well-developed health service delivery infrastructures but face resource constraints; and low-income countries that lack the resources and infrastructure for effective health interventions. Many countries in the region are suffering from political instability, conflicts, and other complex development challenges. This range from low to high incomes can be seen as a microcosm of the variability in maternal mortality present globally. This paper seeks to examine in detail the burden of maternal mortality within the EMR using the results of the GBD 2015 study.

## Methods

A detailed methodology of maternal mortality estimation for GBD 2015 has been published elsewhere (Kassebaum et al. 2016b). In short, sources with population-level data were processed using standardized algorithms to adjust for age-specific, year-specific, and geography-specific patterns of incompleteness. Additional steps were taken to account for underestimations of maternal mortality in vital registration systems. ICD-10 vital registration codes pertaining to HIV-related maternal deaths were excluded and quantified separately (Kassebaum et al. 2014). Overall maternal mortality was modeled using cause-of-death ensemble modeling (CODEm), where all combinations of covariates were tested and ranked on the basis of out-of-sample predictive validity performance. All data sources are available on our website, and we provide a visualization to show each source used in our analyses (Institute for Health Metrics and Evaluation 2016).

We calculated years of life lost (YLLs) by multiplying deaths by the residual expected individual lifespan at the age of death as derived from the GBD 2015 standard model life table. Years lived with disability (YLDs) were calculated by multiplying the number of prevalent cases of a certain health outcome by the disability weight assigned to this health outcome. A disability weight reflects the magnitude of the health loss associated with an outcome and has a value that is anchored between 0, equivalent to full health, and 1, equivalent to death. YLLs were calculated by multiplying deaths by the remaining life expectancy at the age of death from a standard life table chosen as the norm for estimating premature mortality in GBD. Disability-adjusted life years (DALYs) were calculated by adding YLLs and YLDs. Detailed methods on YLLs, YLDs, and DALYs are published elsewhere (Kassebaum et al. 2016a; Vos et al. 2016; Wang et al. 2016).

We evaluated the associations between maternal mortality and socio-demographic status using the socio-demographic index (SDI). SDI is a composite measure developed for GBD 2015 that accounts for fertility rate, lag-dependent income per capita, and education. To capture the average relationships for each age group, we applied a simple least squares spline regression of the maternal mortality rate on SDI. The SDI is scaled from 0 to 1, where 0 represents the lowest possible observed SDI and 1 is the highest. We reported uncertainty for all our estimates (Kassebaum et al. 2016a).

## Results

### Mortality

The total number of deaths due to maternal disorders increased 4.5% from 38,595 (32,859–44,785) deaths in 1990 to 40,338 (31,965–49,954) in 2015 in the EMR, compared to a 29.5% decrease globally (Institute for Health Metrics and Evaluation 2017). In 1990, the maternal mortality ratio was similar in the EMR and globally at 283 (241–328) maternal deaths per 100,000 live births in the EMR and 280 (263–299) globally (Institute for Health Metrics and Evaluation 2017). By 1995, a gap between the regional and global trends had opened, and this persisted steadily through 2015. By then, the EMR had a maternal mortality ratio of 237 (188–293) compared to 194 (173–223) globally (Institute for Health Metrics and Evaluation 2017). By country, only Afghanistan and Djibouti had an annual increase in maternal mortality ratio between 1990 and 2015 (Table 1). Palestine also had an increase in maternal mortality ratio between 2000 and 2015, but still had a lower ratio in 2015 compared to 1990 (Table 1).

**Table 1:** Maternal deaths, maternal mortality ratio, and annualized change in maternal mortality ratio in the Eastern Mediterranean Region, 1990–2015 (Global Burden of Disease 2015 study, Eastern Mediterranean countries, 1990–2015)

Country	Number of maternal deaths			Maternal mortality ratio (per 100,000 livebirths)			Annualized rate of change in maternal mortality ratio (%)		
	1990	2000	2015	1990	2000	2015	1990–2000	2000–15	1990–2015
Afghanistan	4590 (2825–7111)	7328 (4529–11,007)	8525 (5010–13,221)	732.3 (451.0–1130.9)	753.3 (466.5–1130.7)	788.9 (464.1–1219.2)	0.4 (−4.8–5.0)	0.3 (−3.6–3.8)	0.3 (−2.1–2.7)
Bahrain	8 (6–11)	5 (4–7)	5 (4–7)	53.6 (39.9–73.5)	35.6 (27.3–47.3)	24.6 (18.3–35.4)	−4.0 (−7.9 to −0.6)	−2.5 (−5.1 to 0.4)	−3.1 (−4.9 to −1.3)
Djibouti	86 (35–159)	118 (43–283)	107 (43–299)	378.3 (154.4–698.7)	523.8 (192.3–1247.0)	486.2 (193.8–1353.5)	2.8 (−9.8 to 15.3)	−0.5 (−9.4 to 9.9)	0.6 (−4.1 to 6.6)
Egypt	2744 (2308–3176)	1186 (976–1414)	1052 (809–1340)	146.7 (123.4–169.7)	69.2 (57.0–82.5)	42.3 (32.5–53.9)	−7.5 (−9.7 to −5.5)	−3.3 (−5.3 to −1.2)	−5.0 (−6.1 to −3.7)
Iran	1039 (704–1432)	426 (337–539)	281 (192–421)	56.6 (38.5–78.0)	34.5 (27.3–43.7)	20.8 (14.2–31.1)	−5.0 (−8.6 to −0.6)	−3.5 (−6.4 to −0.2)	−4.1 (−6.0 to −1.9)
Iraq	969 (702–1300)	950 (655–1311)	729 (428–1199)	146.0 (105.9–195.9)	113.4 (78.3–156.4)	58.6 (34.4–96.1)	−2.5 (−6.7 to 1.4)	−4.5 (−8.2 to −1.0)	−3.7 (−6.0 to −1.3)
Jordan	116 (89–149)	121 (88–159)	48 (36–63)	97.9 (74.6–125.4)	81.2 (58.7–106.2)	24.2 (17.9–31.5)	−1.9 (−5.1 to 1.1)	−8.0 (−10.7 to −5.4)	−5.6 (−7.2 to −4.1)
Kuwait	4 (3–5)	5 (4–6)	4 (3–6)	9.7 (8.1–11.8)	11.9 (10.4–13.5)	5.9 (4.4–7.5)	2.1 (0.0–4.1)	−4.8 (−6.8 to −2.8)	−2.0 (−3.4 to −0.7)
Lebanon	24 (15–38)	17 (11–24)	13 (8–21)	35.6 (22.8–55.1)	26.7 (18.0–39.0)	15.3 (9.0–24.0)	−2.9 (−7.6 to 1.6)	−3.9 (−7.6 to 0.3)	−3.3 (−6.2 to −0.7)
Libya	34 (24–45)	28 (20–37)	30 (20–43)	26.6 (19.1–36.0)	23.9 (17.2–31.5)	22.8 (15.6–32.9)	−1.0 (−4.8 to 2.3)	−0.3 (−3.5 to 2.8)	−0.6 (−2.6 to 1.3)
Morocco	2441 (1891–3201)	1197 (894–1560)	479 (299–756)	332.7 (258.7–436.1)	192.0 (143.4–249.6)	68.5 (42.8–108.1)	−5.4 (−8.8 to −2.4)	−7.0 (−10.1 to −3.4)	−6.4 (−8.6 to −4.3)
Pakistan	16,973 (13,060–21,189)	22,038 (17,789–26,598)	19,005 (14,012–24,369)	391.5 (301.3–488.4)	498.9 (404.1–601.7)	348.6 (257.2–447.0)	2.4 (−0.3 to 5.5)	−2.4 (−4.7 to −0.3)	−0.5 (−1.9 to 0.9)
Palestine	29 (19–43)	17 (14–21)	25 (17–35)	29.3 (19.0–43.7)	14.2 (11.3–17.8)	16.2 (11.5–23.2)	−7.1 (−11.2 to −3.1)	0.8 (−1.9 to 3.9)	−2.4 (−4.7 to 0.2)
Oman	24 (14–38)	12 (8–18)	13 (9–18)	34.1 (20.0–54.8)	21.4 (14.8–31.7)	15.5 (11.0–21.6)	−4.5 (−8.7 to −0.3)	−2.2 (−5.5 to 1.2)	−3.1 (−5.6 to −0.7)
Qatar	7 (4–10)	7 (5–10)	7 (4–9)	64.0 (42.0–91.5)	59.9 (41.4–84.2)	25.5 (16.4–35.7)	−0.6 (−4.6 to 3.6)	−5.7 (−9.5 to −2.4)	−3.7 (−6.0 to −1.6)
Saudi Arabia	124 (93–162)	101 (86–119)	97 (82–115)	21.3 (16.0–27.9)	17.9 (15.2–21.0)	15.7 (13.3–18.5)	−1.6 (−4.0 to 0.6)	−0.9 (−2.4 to 0.6)	−1.2 (−2.5 to 0.1)
Somalia	2509 (418–6 772)	2899 (489–7449)	3443 (661–10 512)	830.5 (138.5–2193.9)	811.0 (137.5–2077.9)	731.1 (140.5–2218.6)	−0.3 (−11.8 to 10.4)	−0.8 (−8.6 to 8.8)	−0.6 (−6.7 to 6.5)
Sudan	4131 (2736–5570)	4653 (2790–6731)	3941 (2098–5999)	485.5 (321.7–654.4)	414.4 (248.7–598.0)	298.8 (159.2–454.7)	−1.5 (−5.7–1.9)	−2.3 (−5.5 to 1.0)	−2.0 (−4.3 to 0.1)
Syria	560 (421–719)	365 (276–480)	237 (181–309)	125.7 (94.6–161.6)	73.6 (55.6–96.7)	54.1 (41.3–70.5)	−5.3 (−8.1 to −2.6)	−2.0 (−4.7 to 0.5)	−3.4 (−5.0 to −1.8)
Tunisia	183 (143–231)	99 (74–126)	82 (54–115)	85.6 (66.9–108.0)	59.2 (44.3–75.2)	40.6 (26.6–56.9)	−3.7 (−6.6 to −0.8)	−2.6 (−5.5 to 0.2)	−3.0 (−4.9 to −1.4)
United Arab Emirates	15 (9–23)	12 (9–17)	18 (11–28)	31.8 (19.9–48.8)	24.0 (17.0–32.4)	18.0 (10.7–28.3)	−2.6 (−7.3 to 2.0)	−2.0 (−6.1 to 2.0)	−2.3 (−5.3 to 0.4)
Yemen	2559 (1342–3890)	2810 (1509–4648)	2631 (1402–5009)	402.6 (211.8–611.4)	399.6 (215.7–659.8)	307.4 (164.5–583.8)	−0.3 (−4.1 to 4.5)	−1.9 (−5.5 to 2.1)	−1.3 (−3.7 to 1.8)

**Fig. 1:**
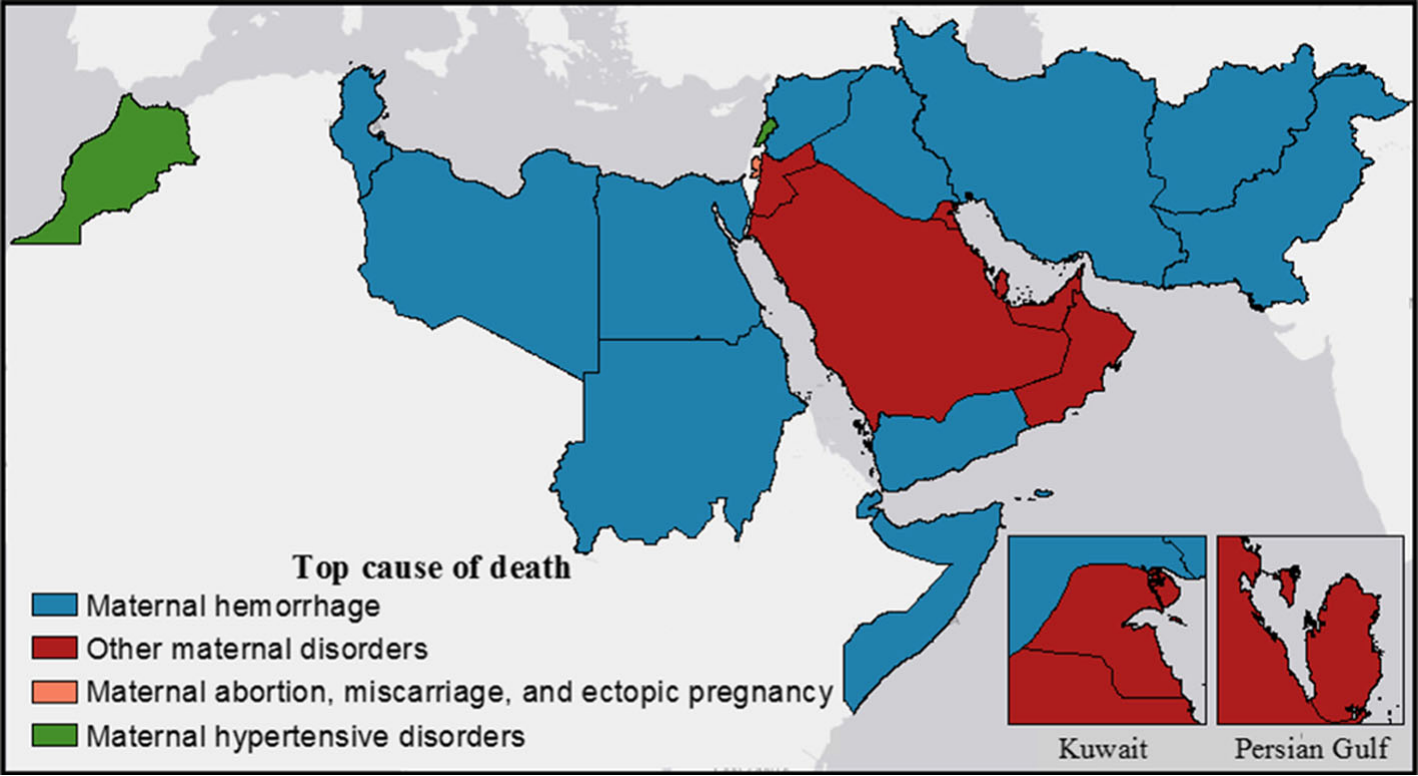
Top cause of maternal deaths in Eastern Mediterranean Region countries, 2015 (Global Burden of Disease 2015 study, Eastern Mediterranean Countries 2015)

Both globally and regionally, maternal hemorrhage was the leading cause of maternal mortality in 1990 and 2015 (Institute for Health Metrics and Evaluation 2017). By country within the EMR, hemorrhage was also the most common, followed by other maternal disorders, noticeably within the Gulf states (Fig. 1). In all countries, all causes of maternal mortality decreased from 1990 to 2015 (Table 2). In 2015, maternal hemorrhage in Somalia was the largest contributor to maternal mortality by country and causes at 72.3 (13.2–229.0) deaths per 100,000 among women aged 15–49 (Table 2). The GBD study expanded the analysis to the full reproductive age range of 10–54 years (Kassebaum et al. 2016b). In general, the countries with the highest mortality rates by cause also showed the least improvement from 1990 to 2015 (Table 2).

**Table 2:** Mortality rates for causes of maternal mortality in the Eastern Mediterranean Region countries, ages 15–49, 2015 and percent change 1990–2015 (Global Burden of Disease 2015 study, Eastern Mediterranean countries, 1990–2015)

Country	Maternal hemorrhage		Maternal hypertension		Other maternal		Complication of abortion	
	Mortality rate per 100,000, 2015	% change, 1990–2015	Mortality rate per 100,000, 2015	% change, 1990–2015	Mortality rate per 100,000, 2015	% change, 1990–2015	Mortality rate per 100,000, 2015	% change, 1990–2015
Eastern Mediterranean Region	7.61 (5.51–10.37)	−52.27	4.49 (3.29–6.11)	−40.39	3.27 (2.37–4.34)	−48.51	2.59 (1.84–3.53)	−50.05
Afghanistan	47.70 (27.17–77.35)	−36.96	20.12 (11.20–32.87)	−29.85	13.99 (7.81 to 23.27)	−33.95	12.82 (7.07–21.40)	−35.04
Bahrain	0.28 (0.19–0.42)	−80.69	0.31 (0.21–0.47)	−73.93	0.43 (0.30–0.65)	−77.48	0.22 (0.15–0.33)	−74.69
Djibouti	17.36 (6.43–49.10)	−30.30	7.26 (2.58–20.67)	−29.47	4.04 (1.39–11.26)	−30.29	3.61 (1.12–9.89)	−30.47
Egypt	1.90 (1.43–2.48)	−81.48	0.76 (0.54–0.99)	−71.80	0.32 (0.24–0.44)	−55.19	0.59 (0.44–0.78)	−73.29
Iran	0.29 (0.19–0.45)	−88.16	0.17 (0.11–0.27)	−81.40	0.13 (0.09–0.21)	−88.48	0.14 (0.09–0.22)	−88.12
Iraq	3.04 (1.71–5.24)	−70.49	1.04 (0.56–1.85)	−61.10	0.91 (0.47–1.63)	−66.49	1.74 (0.96–2.85)	−66.30
Jordan	0.38 (0.25–0.54)	−89.46	0.34 (0.23–0.49)	−83.99	0.99 (0.69–1.34)	−82.45	0.18 (0.12–0.26)	−86.30
Kuwait	0.07 (0.04–0.09)	−52.16	0.04 (0.03–0.06)	−31.67	0.10 (0.07–0.14)	−48.26	0.09 (0.07–0.13)	−44.51
Lebanon	0.1`6 (0.09–0.27)	−80.68	0.18 (0.10–0.29)	−73.17	0.16 (0.09–0.26)	−76.51	0.10 (0.06–0.17)	−78.00
Libya	0.42 (0.26–0.64)	−54.16	0.26 (0.16–0.41)	−45.63	0.34 (0.21–0.53)	−51.63	0.26 (0.15–0.43)	−46.24
Morocco	1.17 (0.71–1.95)	−89.64	1.19 (0.72–1.91)	−85.15	1.06 (0.64–1.70)	−86.86	0.57 (0.34–0.92)	−88.82
Oman	0.18 (0.11–0.28)	−83.85	0.34 (0.22–0.50)	−77.27	0.35 (0.22–0.52)	−80.07	0.25 (0.16–0.36)	−82.47
Pakistan	9.63 (6.44–13.52)	−52.83	8.81 (5.88–12.43)	−35.15	5.54 (3.46–7.77)	−44.39	4.08 (2.57–6.09)	−47.04
Palestine	0.47 (0.30–0.72)	−70.21	0.38 (0.24–0.58)	−59.52	0.34 (0.22–0.53)	−67.21	0.49 (0.32–0.73)	−65.73
Qatar	0.23 (0.13–0.35)	−83.54	0.35 (0.21–0.52)	−76.08	0.38 (0.23–0.57)	−79.73	0.16 (0.09–0.24)	−80.55
Saudi Arabia	0.15 (0.11–0.20)	−74.35	0.09 (0.06–0.12)	−66.88	0.50 (0.40–0.63)	−59.58	0.25 (0.18–0.33)	−68.39
Somalia	72.34 (13.24–229.05)	−17.78	15.14 (2.95–47.52)	−17.10	10.88 (2.01–33.59)	−18.94	11.63 (2.13–35.48)	−17.53
Sudan	8.52 (4.32–13.68)	−64.24	5.09 (2.55–8.46)	−51.00	5.49 (2.83–9.17)	−55.11	3.02 (1.51–4.90)	−53.80
Syria	1.70 (1.18–2.34)	−77.15	0.66 (0.43–0.97)	−69.42	1.17 (0.82–1.62)	−74.60	0.33 (0.22–0.48)	−72.31
Tunisia	1.00 (0.60–1.46)	−72.70	0.36 (0.22–0.55)	−67.89	0.48 (0.30–0.72)	−68.33	0.20 (0.11–0.32)	−67.14
United Arab Emirates	0.18 (0.10–0.31)	−79.78	0.24 (0.13–0.39)	−73.34	0.27 (0.15–0.46)	−78.40	0.18 (0.10–0.30)	−76.09
Yemen	12.07 (6.03–24.02)	−64.23	5.40 (2.66–10.39)	−55.94	7.08 (3.51–14.63)	−60.55	5.48 (2.70–10.85)	−63.71

Country	Maternal indirect		Obstructed labor		Maternal sepsis		Maternal late	
	Mortality rate per 100,000, 2015	% change, 1990–2015	Mortality rate per 100,000, 2015	% change, 1990–2015	Mortality rate per 100,000, 2015	% change, 1990–2015	Mortality rate per 100,000, 2015	% change, 1990–2015
Eastern Mediterranean Region	2.05 (1.40–2.91)	−48.67	1.93 (1.33–2.65)	−38.25	1.86 (1.32–2.57)	−54.00	0.63 (0.37–1.00)	−46.51
Afghanistan	6.94 (3.71–11.87)	−33.85	6.56 (3.57–10.95)	−28.66	4.49 (2.42–7.52)	−34.04	1.82 (0.90–3.29)	−30.65
Bahrain	0.18 (0.12–0.27)	−77.82	0.02 (0.01–0.03)	−74.29	0.09 (0.06–0.13)	−76.25	0.03 (0.01–0.04)	−77.72
Djibouti	5.95 (2.00–16.88)	−30.94	1.36 (0.38–4.09)	−31.64	2.51 (0.80–7.26)	−30.79	1.88 (0.55–5.39)	−26.60
Egypt	0.13 (0.07–0.21)	−83.00	0.16 (0.11–0.23)	−61.01	0.55 (0.39–0.74)	−83.76	0.06 (0.04–0.09)	−77.93
Iran	0.24 (0.16–0.37)	−81.40	0.05 (0.03–0.08)	−85.86	0.11 (0.07–0.17)	−85.22	0.02 (0.01–0.04)	−85.56
Iraq	0.42 (0.21–0.76)	−68.17	0.18 (0.09–0.33)	−56.85	0.67 (0.36–1.20)	−65.77	0.15 (0.07–0.28)	−65.80
Jordan	0.19 (0.12–0.28)	−87.75	0.11 (0.07–0.17)	−84.20	0.20 (0.13–0.29)	−86.93	0.04 (0.03–0.07)	−86.91
Kuwait	0.04 (0.03–0.06)	−43.63	0.01 (0.01–0.02)	−36.29	0.04 (0.02–0.05)	−39.62	0.01 (0.00–0.01)	−43.71
Lebanon	0.08 (0.04–0.14)	−76.84	0.03 (0.01–0.06)	−74.99	0.07 (0.04–0.12)	−76.85	0.02 (0.01–0.03)	−76.89
Libya	0.15 (0.09–0.25)	−55.50	0.06 (0.03–0.10)	−42.48	0.16 (0.09–0.25)	−51.42	0.04 (0.02–0.06)	−49.31
Morocco	0.24 (0.13–0.41)	−86.68	0.34 (0.19–0.59)	−82.51	0.45 (0.27–0.74)	−86.78	0.06 (0.03–0.10)	−87.29
Oman	0.17 (0.11–0.26)	−80.48	0.02 (0.01–0.03)	−78.77	0.07 (0.04–0.10)	−81.47	0.02 (0.01–0.04)	−79.45
Pakistan	2.75 (1.69–4.11)	−44.69	4.84 (3.21–6.91)	−37.12	2.88 (1.77–4.28)	−44.67	1.16 (0.62–1.90)	−45.07
Palestine	0.18 (0.11–0.29)	−67.34	0.04 (0.02–0.07)	−62.46	0.12 (0.07–0.19)	−67.65	0.07 (0.04–0.12)	−64.98
Qatar	0.23 (0.14–0.36)	−81.22	0.02 (0.01–0.03)	−78.54	0.22 (0.14–0.35)	−78.62	0.07 (0.04–0.11)	−74.65
Saudi Arabia	0.07 (0.05–0.10)	−73.74	0.02 (0.01–0.03)	−70.76	0.14 (0.10–0.18)	−70.85	0.02 (0.01–0.03)	−69.15
Somalia	15.86 (2.85–50.91)	−19.42	3.35 (0.53–11.26)	−21.48	6.66 (1.16–21.53)	−20.07	5.38 (0.95–17.98)	−17.88
Sudan	7.44 (3.88–11.88)	−57.43	1.09 (0.54–1.97)	−47.24	6.91 (3.65–11.12)	−52.96	1.09 (0.52–1.95)	−58.23
Syria	0.33 (0.22–0.49)	−75.16	0.28 (0.18–0.43)	−72.32	0.53 (0.36–0.75)	−72.64	0.13 (0.07–0.20)	−73.98
Tunisia	0.17 (0.10–0.27)	−77.38	0.12 (0.06–0.22)	−60.52	0.21 (0.13–0.32)	−72.55	0.07 (0.04–0.13)	−75.21
United Arab Emirates	0.08 (0.04–0.14)	−78.97	0.05 (0.02–0.09)	−73.71	0.04 (0.02–0.07)	−77.92	0.03 (0.01–0.05)	−75.71
Yemen	3.77 (1.79–7.49)	−60.35	1.15 (0.48–2.43)	−55.61	3.28 (1.64–6.14)	−61.43	0.90 (0.42–1.90)	−58.58

### YLLs

YLL rates fell 49% in the EMR between 1990 and 2015, the same as the global mean, from 2,683 (2284–3103) per 100,000 women aged 15–49 to 1377 (1091–1695) (Table 3). In that time frame, the YLL rate decreased in all countries (Table 3). There was significant inter-country variation in YLL rates. The highest YLL rate in 2015 was in Somalia with 7774 (1497–23,408) YLLs per 100,000 women aged 15–49, compared to Kuwait at 21 (16–27) (Table 3). Somalia also had the smallest percent change from 1990 to 2015, a decrease of 18% (Table 3).

**Table 3:** Socio-demographic Index (SDI); years of life lost (YLL), years lived with disability (YLD), and disability-adjusted life years (DALY) rates and percent change; and YLL/YLD ratio for maternal causes in the Eastern Mediterranean Region, women ages 15–49, 1990–2015 (Global Burden of Disease 2015 study, Eastern Mediterranean countries, 1990–2015)

Country	SDI	YLL rate (per 100,000)			YLD rate (per 100,000)			YLL/YLD ratio		DALY rate (per 100,000)		
		1990	2015	% change	1990	2015	% change	1990	2015	1990	2015	% change
Eastern Mediterranean Region	0.55	600 (512–694)	354 (281–435)	−41	39 (27–52)	22 (15–30)	−43	15.38	16.09	639 (549–735)	376 (303–457)	−41
Afghanistan	0.29	2036 (1253–3135)	1431 (842–2218)	−30	26 (17–37)	16 (10–23)	−39	78.31	89.44	2062 (1274–3160)	1446 (856–2233)	−30
Bahrain	0.78	85 (63–116)	19 (14–27)	−78	14 (9–21)	6 (4–10)	−56	6.07	3.17	99 (77–132)	25 (19–33)	−75
Djibouti	0.46	869 (357–1586)	681 (274–1901)	−22	68 (45–98)	48 (31–68)	−30	12.78	14.19	937 (424–1659)	729 (319–1941)	−22
Egypt	0.62	274 (229–319)	65 (50–82)	−76	15 (9–21)	11 (7–17)	−26	18.27	5.91	289 (245–333)	76 (61–94)	−74
Iran	0.72	104 (71–146)	19 (13–29)	−81	15 (9–22)	7 (4–10)	−56	6.93	2.71	119 (84–162)	26 (19–37)	−78
Iraq	0.58	312 (226–417)	109 (63–178)	−65	17 (10–24)	13 (8–20)	−21	18.35	8.38	329 (241–436)	122 (75–190)	−63
Jordan	0.7	196 (149–252)	35 (26–46)	−82	20 (13–30)	14 (8–22)	−31	9.80	2.50	216 (168–271)	49 (37–61)	−77
Kuwait	0.86	10 (9–13)	6 (4–8)	−43	6 (4–9)	5 (3–8)	−9	1.67	1.20	16 (14–20)	11 (9–15)	−30
Lebanon	0.75	51 (33–79)	13 (8–21)	−74	10 (6–15)	5 (3–8)	−47	5.10	2.60	61 (42–90)	18 (13–27)	−70
Libya	0.64	41 (29–56)	24 (16–34)	−42	12 (8–18)	8 (5–13)	−34	3.42	3.00	54 (41–69)	32 (23–43)	−40
Morocco	0.5	541 (418–714)	74 (47–117)	−86	15 (9–22)	9 (5–14)	−41	36.07	8.22	556 (432–730)	83 (54–127)	−85
Oman	0.73	71 (42–113)	15 (11–21)	−78	18 (11–26)	7 (4–12)	−59	3.94	2.14	88 (58–131)	23 (17–30)	−74
Pakistan	0.47	910 (696–1142)	581 (426–744)	−36	74 (50–102)	41 (28–57)	−45	12.30	14.17	984 (777–1213)	622 (469–784)	−37
Palestine	0.57	76 (50–113)	29 (21–43)	−62	19 (12–28)	12 (7–18)	−38	4.00	2.42	95 (66–132)	41 (32–55)	−57
Qatar	0.8	77 (51–110)	16 (11–23)	−79	10 (6–15)	4 (3–7)	−55	7.70	4.00	87 (60–120)	21 (14–27)	−76
Saudi Arabia	0.76	42 (32–55)	16 (14–19)	−61	16 (10–24)	8 (5–12)	−49	2.63	2.00	58 (46–72)	25 (20–30)	−58
Somalia	0.15	2144 (361–5693)	1741 (341–5215)	−19	102 (68–149)	61 (42–87)	−40	21.02	28.54	2246 (464–5797)	1802 (409–5273)	−20
Sudan	0.43	1159 (758–1561)	526 (284–785)	−55	43 (28–64)	26 (17–36)	−41	26.95	20.23	1202 (803–1602)	552 (306–810)	−54
Syria	0.58	264 (199–341)	74 (56–97)	−72	17 (11–26)	10 (6–16)	−42	15.53	7.40	281 (216–357)	84 (65–106)	−70
Tunisia	0.65	128 (100–163)	39 (26–55)	−69	11 (7–17)	7 (4–11)	−36	11.64	5.57	140 (110–175)	47 (33–63)	−67
United Arab Emirates	0.88	44 (28–68)	10 (6–16)	−77	11 (7–16)	3 (2–5)	−72	4.00	3.33	55 (37–79)	13 (9–19)	−76
Yemen	0.41	1235 (656–1865)	562 (304–1058)	−55	104 (73–141)	42 (29–59)	−60	11.88	13.38	1339 (754–1982)	604 (347–1103)	−55

### YLDs

Average YLD rates in the region fell from 161 (114–215) per 100,000 women aged 15–49 to 82 (57–113), a 49% decrease (Table 3). Globally, there was a 41% decrease (Institute for Health Metrics and Evaluation 2017). Inter-country variability in the YLD rate was less than that of YLLs, with the highest seen in Somalia with 254 (171–362) YLDs per 100,000 women aged 15–49 and the lowest in the United Arab Emirates with 16 (10–26) (Table 3).

### YLL/YLD

The YLL/YLD ratio was included as an indicator of health system effectiveness in dealing with fatal and non-fatal outcomes of maternal disorders. Large values, such as that of Afghanistan, a ratio of 89.6 in 2015, demonstrated cases, where fatal outcomes heavily outweigh non-fatal ones (Table 3). Kuwait in 2015 had a ratio of 1.11, signifying a near equal burden of fatal and non-fatal outcomes for maternal disorders (Table 3). Lower income countries with SDI less than 0.5 all had ratios greater than 10, whereas higher income countries with SDI greater than 0.7 had ratios under 4 (Table 3). In the countries with SDI less than 0.5, all but Sudan had an increase in YLL/YLD ratio from 1990 to 2015 (Table 3). Large decreases in the ratio from 1990 to 2015 highlighted effective improvements in treatment, such as in Morocco, where the ratio decreased from 35.2 to 8.5 (Table 3).

### DALYs

**Fig. 2:**
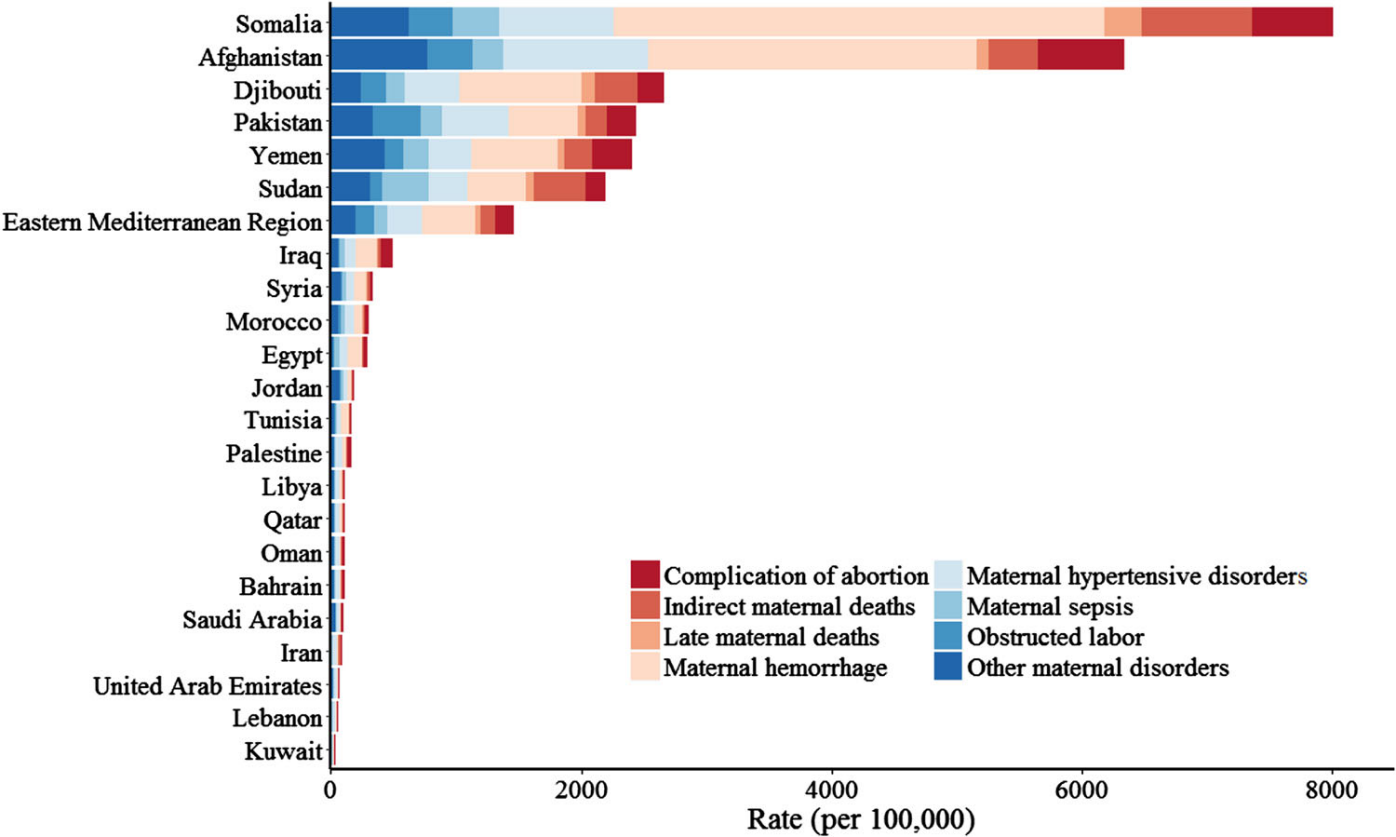
Disability-adjusted life-year (DALY) rates per 100,000 women ages 15–49 for maternal causes by country, 2015 (Global Burden of Disease 2015 Study, Eastern Mediterranean Countries 2015)

**Fig. 3:**
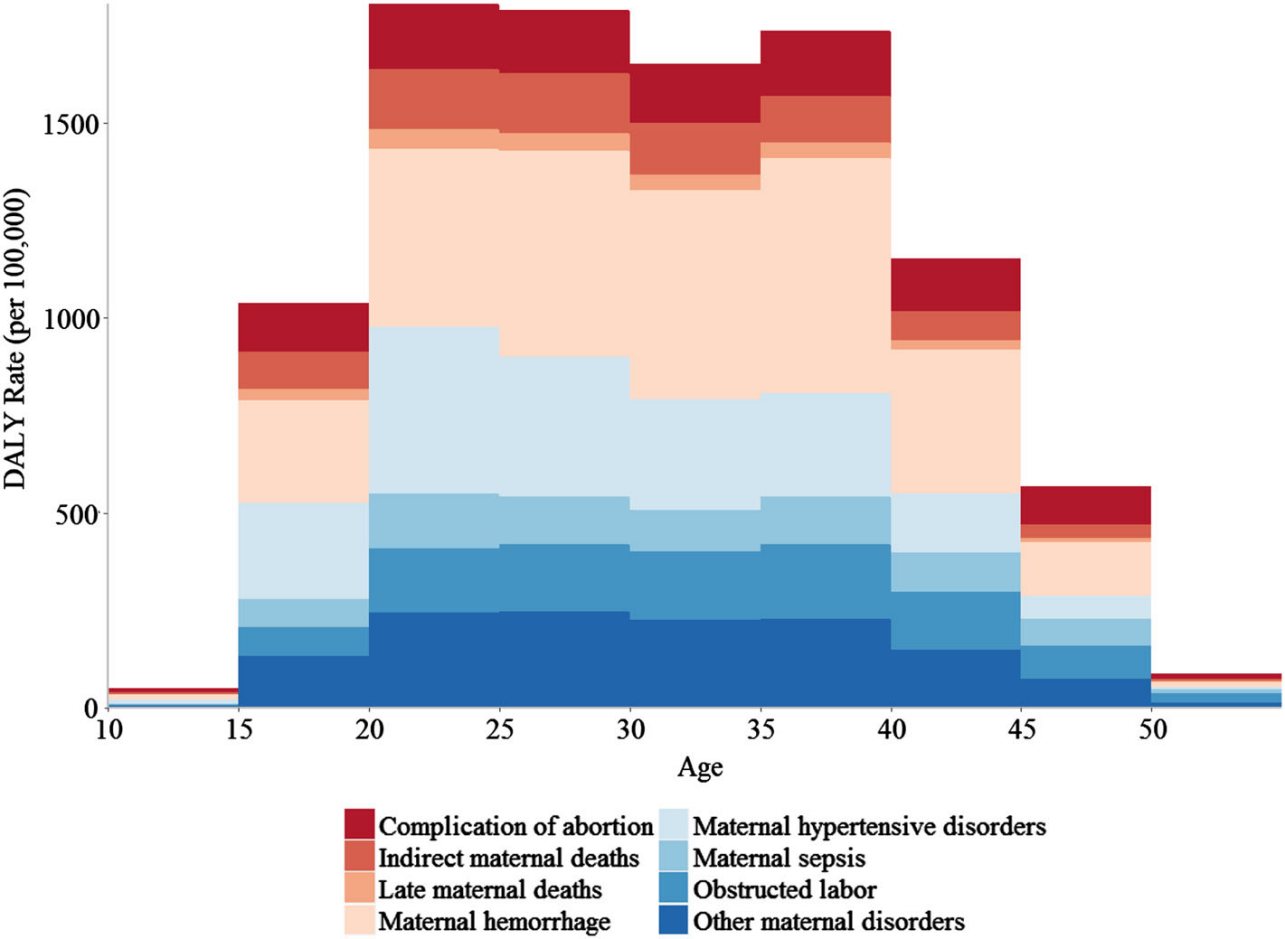
Disability-adjusted life-year (DALY) rates per 100,000 women ages 10–54 for maternal causes in the Eastern Mediterranean Region by age, 2015 (Global Burden of Disease 2015 study, Eastern Mediterranean Region 2015)

From 1990 to 2015, DALY rates decreased in all countries in the region. The overall DALY rate for the region decreased by 49% during this interval, compared to 51% globally (Institute for Health Metrics and Evaluation 2017). As discussed in the YLL/YLD section above, the proportional contribution of YLDs to the DALYs rate increased as SDI increased (Table 3). Like YLL rates, DALY rates showed large inter-country variation, as shown in Fig. 2. DALY rates peaked in the 20–25-year age group, decreasing until ages 35–40, then steeply falling off in 5-year increments (Fig. 3). By sub-cause, complication of abortions contributed the most to the DALY rate in young (10–15) and old (45+) age groups (e-Fig. 1). Percent of DALYs attributable to obstructed labor increased steadily with age, spiking drastically for the 50–55-year age group (e-Fig. 1).

## Discussion

Our study showed that maternal mortality has declined in the EMR except in Djibouti, Palestine, and Afghanistan in recent years. However, the burden is still higher than the global average. Our findings call for increased efforts to reduce maternal mortality in the region. Moreover, with unrest in the region, there is a risk of losing some of the observed gains.

Efforts to reduce maternal mortality in the EMR have varied in scope, approach, and success by country. This has resulted in great variations and disparities in maternal mortality levels between countries. The high-income countries in the region (Kuwait, Oman, Qatar, Saudi Arabia, and United Arab Emirates) have achieved reductions between 25 and 50% compared to levels in 1990. Maternal mortality ratios in these countries ranged from 6 to 26 deaths per 100,000 live births, consistent with countries of similar income around the world. Certain middle-income countries such as Morocco and Jordan have successfully implemented interventions that have drastically reduced maternal mortality by greater than 75% from 1990 levels. Low-income countries have seen the least improvement, or in some cases none at all. Reduction in maternal mortality in Pakistan, Somalia, Sudan, and Yemen ranged from 11 to 39%, and maternal mortality in Afghanistan and Djibouti increased from 1990 to 2015. Maternal mortality ratios for these countries ranged from 299 deaths per 100,000 live births in Sudan to 789 in Afghanistan. This discussion will examine the obstacles to effective maternal care, the direct and underlying causes of maternal mortality, and the challenges faced by countries in the region to improve maternal health.

The process of improving maternal health in the region faces many challenges which have slowed progress in recent years. For many years, the EMR has been devastated by manmade disasters and conflicts, which have destroyed infrastructure in several nations and have tremendously affected the health of the population, especially for vulnerable groups such as mothers and children. The humanitarian community has made several efforts to address these challenges, and these have reduced the potential impact of the unrest. However, the disruption of health care systems has had a negative impact. The social and health impacts of political instability, domestic crises, and economic sanctions are well-documented in the region (Mokdad et al. 2016). Recent distress in the region will result in deteriorating health conditions in these countries for many years to come. Despite this, however, the region has experienced improved health and life expectancy.

The region is in need of efforts to improve preventive strategies for reducing maternal mortality. A literature review (Warren et al. 2015) that evaluated the evidence on the effectiveness of sexual and reproductive health interventions delivered in humanitarian crises found some evidence to support increased access and demand creation for family planning services through community health workers, health care subsidies, and discussions within literacy groups. Involving communities in maternal and child health and birth preparedness programs, as well as refurbishing clinics and hospital facilities, was also associated with increased positive health outcomes (Warren et al. 2015).

Female genital mutilation (FMG) is still practiced in many countries in the EMR due to a mix of sociocultural factors within families and communities (UNICEF 2016). Experience of FGM increases the short- and long-term health risks to women and young girls between infancy and age 15, and is unacceptable from a human rights and health perspective. Procedures can cause severe bleeding and problems urinating, and later, cysts, infections, as well as complications in childbirth and increased risk of newborn deaths (WHO 2017).

Thaddeus and Maine provide a conceptual model for understanding factors that obstruct favorable obstetric outcomes that consists of three types of delays a pregnant woman faces when seeking care—delays in deciding to seek care, in reaching an adequate facility, and in receiving adequate care in a facility. The first delay manifests itself in the way women and their families perceive the accessibility of services, often shaped by prior experiences with health facilities. Any number of factors may influence a decision to delay treatment, such as cost, distance, lack of proper medical supplies, and unhelpful or impolite staff. The second delay accounts for the time taken to reach a medical facility. This is a particularly large obstacle for rural areas in which the availability of transportation is uncertain or expensive. In addition, data on maternal deaths that occur on the way to a health facility are scarce. The third and final delays are in receiving adequate care at health facility, which can be attributed to insufficient staff, drugs, and equipment. It is necessary for interventions to effectively address the underlying causes of these delays on a country-by-country basis (Thaddeus and Maine 1994).

This conceptual model is backed by an extensive body of research on the direct and underlying causes of maternal mortality. Improving women’s literacy and knowledge can significantly contribute to their health. Alvarez et al. reported a significant negative relationship between MMR and gross domestic product (GDP) per capita, health expenditure (HE), women’s literacy rate, and the number of deliveries by skilled birth attendants in the study region in Africa (Alvarez et al. 2009). Another study in sub-Saharan African countries, concluded that birth in the presence of health professionals and the life expectancy at birth are highly correlated to maternal mortality. Furthermore, a convincing relationship was found between GDP per capita and health expenditure per capita and maternal mortality (Buor and Bream 2004). Similarly, a study in Pakistan reported that a significant relationship was observed between having access to health services, professional health staff, and health care during pregnancy and reductions in MMR (Midhet et al. 1998). The positive impacts of GDP, HE, and female education level on health outcomes were also reported in other studies in the EMR (Bayati et al. 2013; Homaie Rad et al. 2013).

Success stories do exist in the region: for example, Morocco was one of only ten countries globally that met the MDG 5 requirement of an annual rate of decline exceeding 5.5% for maternal mortality ratio every year from 1990 to 2015 (Kassebaum et al. 2016b). This improvement can be traced to a number of programs implemented to improve determinants of health and health coverage. Increases in the number of health facilities and training programs led to the percentage of deliveries assisted by skilled personnel increasing from 31% in 1992 to 73.5% in 2011. Other successes in the region occurred in Jordan and Iran, where maternal mortality was reduced by more than 50% from levels in 1990 (Kassebaum et al. 2016b).

Uneven improvements in MMR in the region may be related to differing levels of ramp-up in coverage for specific types of reproductive health care—antenatal care, in-facility delivery, skilled birth attendance, family planning services, emergency obstetric care, and post-natal care—that are all known to decrease the risk of negative pregnancy outcomes (Lim et al. 2010; Randive et al. 2014). Quality of care must also be considered during buildup of the maternal health care system. Programs for reproductive health care must ensure that women are receiving the care they need during pregnancy and the post-partum period (Rowe et al. 2005). Care should be integrated and not be focused on single vertical interventions (Campbell et al. 2006).

It has been challenging for many countries to produce timely and accurate data on levels of maternal mortality that would indicate the extent of their progress in reducing maternal deaths (Mokdad et al. 2016). Reliable information is a necessary component of any strategy aimed at reducing maternal mortality. Continued progress in data collection in the EMR is key to evaluating progress in reducing maternal mortality.

Our study has several limitations. First, many countries in the region have poor health data and vital statistics. We used GBD methodology to account for quality and lack of data. We also applied our standard GBD garbage code correction to address this limitation. Second, little information is available on unsafe abortion in the region due to religion and culture. However, our study is the most comprehensive on burden of diseases and applies the standard methodology that allows global comparison.

## Conclusion

Progress in reducing maternal mortality in the EMR has accelerated in the past 15 years, but there is still much to do to reduce preventable deaths. Extending basic maternal health services, improving quality of care, and eliminating unmet need for contraception are all crucial, proven steps effective at reducing MMR. Our study showed the importance of empowering women: increased women’s rights are needed to improve their health. Finally, coordinated and rigorous efforts are needed to make sure that every woman in need receives these interventions in a timely fashion at each stage of her reproductive life.

## Electronic supplementary material

Below is the link to the electronic supplementary material.

Supplementary material 1 (DOCX 26 kb)

Supplementary material 2 (XLSX 30 kb)
