# Supplementary material for: Maternal mortality and morbidity burden in the Eastern Mediterranean Region: findings from the Global Burden of Disease 2015 study
Source: Int J Public Health. 2017 Aug 3;63(Suppl 1):47–61. doi: 10.1007/s00038-017-1004-3 (PMC5973988; doi:10.1007/s00038-017-1004-3)

Electronic Supplementary Material

**Article title:**

Maternal mortality and morbidity burden in the Eastern Mediterranean Region: Findings from the Global Burden of Disease 2015 study

**Journal:**

International Journal of Public Health

**Authors:**

GBD 2015 Eastern Mediterranean Region Maternal Mortality Collaborators

**Corresponding author:**

Ali H. Mokdad

Institute for Health Metrics and Evaluation, University of Washington, Seattle, WA, United States

Email: [mokdaa@uw.edu](mailto:mokdaa@uw.edu)

e-Figure 1 - Percent of total maternal disability-adjusted life-years(DALYs) attributable to maternal causes in the Eastern Mediterranean by age, 2015. (Global Burden of Disease 2015 Study, Eastern Mediterranean Countries, 2015).


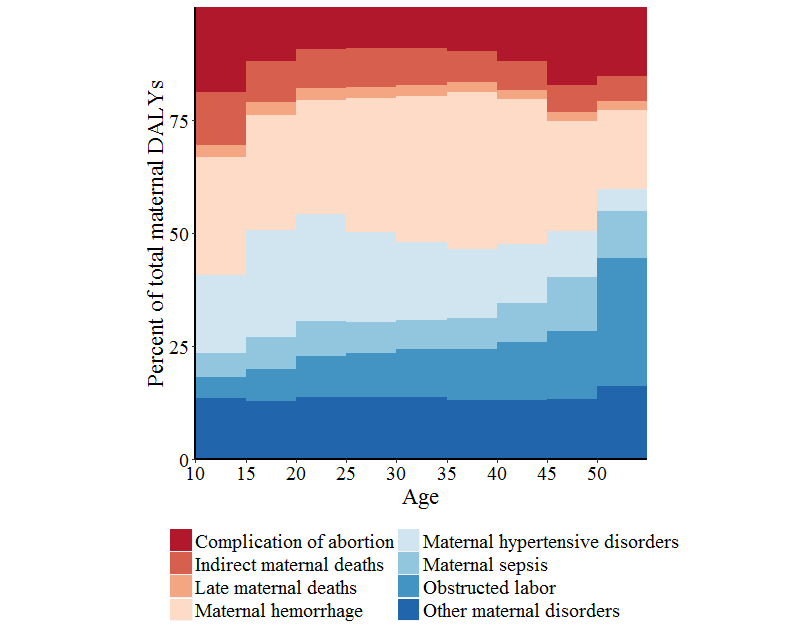

Supplement: Supplementary file 1 — Supplementary material 1 (DOCX 26 kb) [file 38_2017_1004_MOESM1_ESM.docx]
